# Comparison of CDC and sequence-based molecular typing of syphilis treponemes: *tpr* and *arp* loci are variable in multiple samples from the same patient

**DOI:** 10.1186/1471-2180-13-178

**Published:** 2013-07-30

**Authors:** Lenka Mikalová, Petra Pospíšilová, Vladana Woznicová, Ivana Kuklová, Hana Zákoucká, David Šmajs

**Affiliations:** 1 Department of Biology, Faculty of Medicine, Masaryk University, Brno, Czech Republic; 2 Department of Medical Microbiology, Faculty of Medicine, Masaryk University, Brno, Czech Republic; 3 Department of Dermatology, 1st Faculty of Medicine, Charles University in Prague, Prague, Czech Republic; 4 National Reference Laboratory for Diagnostics of Syphilis, The National Institute for Public Health, Prague, Czech Republic

**Keywords:** *Treponema pallidum*, Syphilis, Molecular typing, Sequence-based typing, CDC typing, *arp* gene, *tpr* genes

## Abstract

**Background:**

Molecular typing of syphilis-causing strains provides important epidemiologic data. We tested whether identified molecular subtypes were identical in PCR-positive parallel samples taken from the same patient at a same time. We also tested whether subtype prevalence differs in skin and blood samples.

**Results:**

Eighteen syphilis positive patients (showing both positive serology and PCR), with two PCR-typeable parallel samples taken at the same time, were tested with both CDC (Centers for Disease Control and Prevention) and sequence-based typing. Samples taken from 9 of 18 patients were completely typed for TP0136, TP0548, 23S rDNA, *arp*, and *tpr* loci. The CDC typing revealed 11 distinct genotypes while the sequence-based typing identified 6 genotypes. When results from molecular typing of TP0136, TP0548, and 23S rDNA were analyzed in samples taken from the same patient, no discrepancies in the identified genotypes were found; however, there were discrepancies in 11 of 18 patients (61.1%) samples relative to the *arp* and *tpr* loci. In addition to the above described typing, 127 PCR-positive swabs and whole blood samples were tested for individual genotype frequencies. The repetition number for the *arp* gene was lower in whole blood (WB) samples compared to swab samples. Similarly, the most common *tpr* RFLP type “d” was found to have lower occurrence rates in WB samples while type “e” had an increased occurrence in these samples.

**Conclusions:**

Differences in the CDC subtypes identified in parallel samples indicated genetic instability of the *arp* and *tpr* loci and suggested limited applicability of the CDC typing system in epidemiological studies. Differences in treponemal genotypes detected in whole blood and swab samples suggested important differences between both compartments and/or differences in adherence of treponeme variants to human cells.

## Background

Syphilis, caused by *Treponema pallidum* ssp. *pallidum* (*T*. *pallidum*), is a sexually transmitted multistage disease with a diagnosis based on clinical symptoms, serological findings and other methods such as direct detection of treponemes by microscopy. In the 1990s, PCR-based methods for direct detection of treponemal DNA were developed [1]. Since then, several improvements in these tests have been published which have increased sensitivity and specificity [2-9] as well as the ability to detect the presence of several pathogens simultaneously in the same reaction using multiplex PCR [10,11]. A major advantage of PCR–based methods in syphilis diagnostics is the potential for subsequent molecular typing of syphilis treponemes. Although several treponemal genomic loci were tested relative to their suitability for molecular typing [12-14], most molecular typing studies of treponemal DNA are performed using CDC typing [15]. The method involves detection of the number of 60-bp tandem repeats in the *arp* (acidic repeat protein) gene and restriction fragment length polymorphism (RFLP) analysis of the 3 PCR-amplified *tpr* genes (*tprE*, *G*, *J*). In 2010, the CDC method was modified by addition of sequencing of TP0548 [14] or by determination of the number of G repeats within the *rpsA* gene (TP0279) [16].

Recently, a sequencing-based molecular typing scheme based on sequencing of the TP0136, TP0548 and 23S rRNA genes was introduced [17]. Moreover, the sequence variants of TP0136, TP0548 and 23S rRNA genes have been shown to independently combine with variants of the *arp* and *tpr* genes [17].

In this communication, we compare CDC typing with sequence-based molecular typing in a group of patients with two or more parallel samples (i.e. taken at the same time) that were PCR-positive for treponemal DNA. Moreover, the variability of gene sequences, length and RFLP genotypes are compared in two types of clinical specimens (i.e. swab and whole blood samples).

## Results

### PCR detection and molecular typing of syphilis treponemes

A total of 18 patients had two PCR-typeable samples (taken at the same time), which were used for amplification of treponemal TP0136, TP0548, 23S rRNA genes, *tpr* and *arp* loci. Two separate PCR-typeable swabs were available for each of 9 patients in one group of patients, while a second group of 9 patients had a single PCR-typeable swab along with a whole blood sample. Four patients were diagnosed with secondary syphilis while the remaining 14 patients had primary syphilis (Table 1).

**Table 1:** Molecular typing of treponemal DNA isolated from a set of 16 patients with two or more samples positive for treponemal DNA

**Patient no.**	**Syphilis stage**	**Sample**	**Material**	**Genotypes based on TP0136, TP0548, 23S rDNA**	**CDC subtype**	Enhanced CDC subtype*
1	Primary	43K StI	Swab	SSS	14d	14d/f
		44K StII	Swab	SSS	14d	14d/f
2	Primary	36K St	Swab	SU2R8	14d	14d/g
		37K	Whole blood	SU2R8	12d	12d/g
3	Primary	93K	Swab	SXS	14k	14k/X
		94K	Whole blood	XSX	14e	14e/f
4	Primary	891	Swab	SSS	14d	14d/f
		892	Swab	SSS	14p	14p/f
5	Primary	91K	Swab	SSR8	14d	14d/f
		92K	Whole blood	SXX	12e	12e/X
6	Primary	C-150	Swab	U1SS	8b	8b/f
		D-151	Swab	U1SS	8d	8d/f
7	Primary	8032	Swab	SSR9	15d	15d/f
		8284	Swab	SSR9	15d	15d/f
8	Primary	19512	Swab	SU2R8	14d	14d/g
		19527	Swab	SU2R8	14b	14b/g
9	Primary	15K St	Swab	SSS	14d	14d/f
		16K	Whole blood	XSS	XXe	XXe/f
10	Primary	RL104BZ	Swab	SU2R8	14d	14d/g
		RL104AZ	Whole blood	XU2R8	14X	14X/g
11	Primary	63K	Swab	SSR9	15d	15d/f
		62K	Whole blood	XSR9	15d	15d/f
12	Primary	4K	Swab	SSS	14d	14d/f
		5K	Swab	SSS	14d	14d/f
13	Primary	34K St	Swab	SSS	14d	14d/f
		35K	Whole blood	XXS	XXe	XXe/X
14	Primary	RL116A	Swab	SU2R8	14e	14e/g
		RL116B	Whole blood	SXR8	14j	14j/X
15	Secondary	15577	Swab	SXS	14d	14d/X
		15578	Swab	SSS	14d	14d/f
16	Secondary	G-269	Swab	SU1S	14p	14p/f
		H-270	Swab	SU1S	14d	14d/f
17	Secondary	51K	Swab	SSS	14d	14d/f
		52K	Swab	SSS	14d	14d/f
18	Secondary	73K	Swab	SU2R8	14d	14d/g
		74K	Whole blood	XXR8	XXe	XXe/X

X, the exact genotype was not determined.

*subtype identification based on CDC typing enhanced by sequence analysis of TP0548 between position 131–215 [14].

The samples from these 18 patients were typed with both CDC and sequence-based typing schemes [15,17], the results are shown in Table 1. Samples taken from 9 of 18 patients were completely typed at all loci (TP0136, TP0548, 23S rDNA, *arp*, and *tpr*). The remaining 9 patient samples were partially typed (10 samples were partially typed at the TP0136, TP0548, and 23S rDNA loci and 4 samples were partially typed at the *arp* and *tpr* loci). CDC typing revealed 11 distinct genotypes while sequence-based typing revealed 6 genotypes. The identified sequences of TP0136, TP0548 and 23S rDNA loci are shown in Additional file 1. Using enhanced CDC typing [14], 13 different genotypes were found (Table 1). When results of molecular typing of TP0136, TP0548, and 23S rDNA were available, no discrepancies in the genotypes were identified in samples taken from the same patients (Table 1). In contrast, samples taken from 11 of 18 patients (61.1%) revealed discrepancies at the *arp* and *tpr* loci (1 for *arp*, 9 for *tpr* and 1 for both of these loci). The most frequent discrepancies involved the “d” and “e” (4 cases), “d” and “b” (2 cases) and “d” and “p” (2 cases) patterns of the *tpr* genes. Two of four patients with secondary syphilis had differences at the *tpr* loci (Table 1).

When analysis of loci used in sequence-based (i.e. analysis of TP0136, TP0548 and 23S rDNA) and CDC typing (i.e. *arp* and *tpr* genes) was performed independently, 14 swab/blood paired DNA samples were analyzed in both sequence-based typing and in CDC typing. While no discrepancies were found in sequence-based typing, 8 out of 14 genotypes detected in CDC typing were different. Similarly, analysis of parallel swabs revealed 26 and 18 typed DNA samples for sequence-based and CDC typing, respectively. No discrepancies were found in sequence-based typing while 4 out of 18 genotypes detected in CDC typing were different.

Four of 9 (44.4%) patients (with two positive swabs for treponemal DNA) showed differences in *tpr* gene patterns while 7 of 9 (77.8%) patients (with swabs and whole blood samples) showed pattern differences at the *arp* or *tpr* loci. The 2 differences found in the *arp* gene were found in patients with both swab and whole blood samples and in both cases the repetitions number of the arp gene was lower in whole blood samples compared to swab samples.

### Variability of treponemal genotypes found in whole blood and swab samples

To test whether individual genotype rates differ in swabs vs. whole blood samples, the occurrence rates of individual genotypes was determined in swabs and whole blood samples (Table 2) using the data set from Flasarová et al. [17] augmented by samples collected in 2011 in the Czech Republic. Altogether, 93 swabs and 34 whole blood samples were analyzed. Among the investigated strains, similar proportions of sequences (i.e. SS14-like and unique) were identified for loci TP0136 and TP0548. Similarly, both A2058G and A2059G mutations in the 23S rDNA showed similar occurrence rates in swabs and whole blood samples (Table 2). However, the number of repetitions in the *arp* gene showed a significant difference between swab and WB samples. The *arp* gene with a lower number of repetitions was found to occur more often in WB samples. In addition, the most common *tpr* RFLP type “d” occurred less often in WB samples while type “e” had a higher occurrence rate in WB samples.

**Table 2:** Genotypes identified in PCR positive swabs and whole blood samples

**Genes**		**Type of sample**		**Statistical significance**
	**Locus nucleotide sequences**	**Swabs (n = 93)**	**WB samples (n = 34)**	
**TP0136**	Identical to strain SS14	96.1% (74/77)	100.0% (12/12)	
	Unique sequences	3.9% (3/77)	0.0% (0/12)	
**TP0548**	Identical to strain SS14	74.4% (58/78)	68.8% (11/16)	
	Unique sequences	25.6% (20/78)	31.3% (5/16)	
**23S rDNA**	wt	60.4% (55/91)	51.6% (16/31)	
	A2058G	28.6% (26/91)	25.8% (8/31)	
	A2059G	11.0% (10/91)	22.6% (7/31)	
	**Number of repetitions in the *arp *locus**			
		Swabs	WB samples	
***arp***	14	82.4% (56/68)	55.6% (5/9)	p = 0.03
	15	11.8% (8/68)	11.1% (1/9)	
	8-12	5.9% (4/68)	33.3% (3/9)	p = 0.003
***tpr E, G, J***	***tpr E, G, J *pattern after *Mse*I digest**			
		Swabs	WB samples	
	d	91.2% (62/68)	30.8% (4/13)	p < 0.001
	e	1.5% (1/68)	46.2% (6/13)	p < 0.001
	b, p, k, j	7.4% (5/68)	23.1% (3/13)	

Samples isolated in the work of Flasarová et al. [17] augmented by samples collected in 2011 in the Czech Republic were analyzed. Results show both paired and unpaired samples.

wt, wildtype.

## Discussion

Molecular detection of treponemal DNA and the subsequent molecular typing of *T*. *pallidum* strains have allowed epidemiological mapping of treponemal syphilis strains [15]. In recent years, there has been increasing evidence showing differences in molecular genetic markers among virulent treponemal strains isolated in different countries [14,16-34]. Some studies have shown that predominant treponemal strains in a particular population can change over time [14,17].

The selection of suitable genetic loci appears to be of enormous importance. Genetic loci suitable for molecular typing should contain a relatively high degree of variability and relatively high stability in future generations of the microbial population. Several genetic loci including *tprK*, *tprC* and the intergenic region between TP0126-TP0127 have been tested for their suitability for molecular typing and rejected because of multiallelic sequences [12] or because of a lack of discriminatory power [14].

The most widely used molecular typing system [15] and its improved versions [14,16] are in principle based on detection of genetic variability in the *arp* and *tpr* genes. As shown by Liu et al. [35], the repeat motifs in the *arp* gene code for highly immunogenic protein sequences and represent a potential fibronectin-binding domain. The *arp* gene in *T*. *pallidum* strains is subject to positive selection and the size variation in repeat motifs in *T*. *pallidum* strains is likely connected with mechanisms that treponemes use to escape/evade the host’s immune response, which has been primed against the standard (and the most prevalent repeat number among clinical samples) 14-repeat variant [36]. Genes *tprE*, *G* and *J* are potential virulence factors and belong to *tpr* subfamily II [37]. These genes are expressed during syphilis infection [38,39] and the TprEJ proteins are likely located on the outer membrane [40,41]. Recently, Giacani et al. [40] demonstrated how the number of poly-G repeats effected transcription of *tprE*, *G*, and *J* through a phase variation mechanism, and the modulating effect of the TP0262 gene on the level of transcription of these *tpr* genes [42]. We have shown that these loci are often variable in samples taken from the same patient. Although coinfection with two genetically different treponemal strains cannot be excluded, it appears unlikely since typing with other markers, including the TP0136, TP0548, and 23S rDNA loci, revealed identical sequences despite the fact that the latter typing scheme identified only 6 different genotypes among the 18 patients sampled in this study.

Under experimental conditions, Pillay et al. [15] showed that the CDC genotype is stable in repeated rabbit passages of *T*. *pallidum* Nichols strain and others have confirmed this finding [14]. Moreover, genetic stability has been shown for two additional treponemal strains (Sea 81–4 and Chicago C) using experimental infections of rabbits [14]. However, human infection may differ considerably from experimental rabbit infections. These differences represent differences in IL-2 levels produced by Th1 cells (Helper T cells) during the early cellular response to *T*. *pallidum* in the rabbit model, where the mRNA IL-2 levels were considerably lower than IL-10 levels [43]. On the other hand, IL-2 mRNA levels in early human lesions had comparable levels of IL-10 [44]. Moreover, in contrast to rabbit infections, CD8+ T-cells are often the dominant T-cell during human infections [45].

It has been shown that skin and blood represent two immunologically distinct compartments with respect to syphilis infections [45]. Cellular immunity seems to be more important than humoral immunity in the clearance of *T*. *pallidum* from early syphilis lesions [46]. The inability of humoral immunity to control the infection is demonstrated by formation of secondary syphilis lesions despite the presence of high antibody titers against treponemal antigens [44]. It is likely that these immunologically different compartments produce different selection forces that act on treponemes living in skin lesions and in whole blood. To confirm this hypothesis, we tested a spectrum of different genotypes from both swabs and whole blood samples. Interestingly, the spectrum of the *arp* and *tpr* variant significantly differed between swabs and whole blood samples indicating their instability and differences in selection of treponeme variants in both niches. Alternatively, differences in the *arp* and *tpr* loci could result in lowered adherence of these treponemes to human cells prompting increased migration of these treponemes from primary lesions to other human compartments.

There are only a few studies describing the genetic analysis of multiple parallel samples taken from one patient at the same time [24,34]. Moreover, only a limited number of parallel samples were analyzed in these studies (i.e. involving 2–4 patients) and this fact likely precluded identification of the variability of detected genotypes. In addition, only a limited number of studies used whole blood samples for molecular typing, mainly because of lower frequency of PCR-positive results [18,47-49]. When the published data were analyzed [15,16,18-20,22,24-26,29-32], 19 WB and 536 swab samples were fully typed using the CDC typing system. The most prevalent subtype in swab samples was 14d (351 samples, 65.5%), which were similar to our results (14d subtypes in 50 out of 68 fully typed swabs, 73.5%). As with swabs, the 14 repetition version of the *arp* gene was also the most common in WB samples. The most common *tpr* profile in WB samples was ‘a’, found in 17 of 19 WB samples [18,22]. Interestingly, none of the WB subtypes identified in our study (12d, 12e, 14e, 14j, 14k, 15d) were similar to the published WB subtypes.

There are several limitations to this study. One of these is the small number of available parallel PCR-typeable samples taken from the same patient. Therefore, observed differences should be interpreted with caution and more parallel samples need to be tested in future. Another limitation is the small number of fully-typed samples, especially in the sequence-based typing system. The observed lower discriminatory power of sequence-based typing compared to CDC typing is likely a result of genetic variability of *tpr* and *arp* loci, however, this explanation needs to be verified.

Taken together, parallel samples taken from the same patient, at the same time, revealed potential instability at the *tpr* and *arp* loci, which is often used in molecular typing of treponemes. These loci are likely to show treponemal intra-strain variability and the results of molecular typing should be interpreted with caution, especially in epidemiological studies. Differences in frequencies of genotypes in whole blood and swab samples suggest an antigenic/adherence character for proteins encoded by these loci and also immunological differences between compartments (i.e. skin and whole blood).

## Conclusions

The CDC typing scheme revealed subtype differences in parallel samples taken from 11 of 18 tested patients (61.1%). The *arp* and *tpr* loci are likely to show treponemal intra-strain variability since the sequence-based typing system revealed identical sequences in the TP0136, TP0548, and 23S rRNA genes. Therefore, the results of CDC typing should be interpreted with caution, especially in epidemiological studies. Differences in treponemal genotypes detected in whole blood and swab samples suggest immunological differences between the skin and whole blood compartments and/or differences in adherence of genetic variants of treponemes to human cells.

## Methods

### Collection of clinical samples

Clinical samples were collected from 2006 – 2012 in several clinical departments in the Czech Republic (Department of Medical Microbiology and Department of Dermatology, Faculty of Medicine, St. Anne’s Hospital and Masaryk University Brno, Department of Dermatology, Faculty Hospital Brno, Department of Dermatology, 1st Faculty of Medicine, Charles University in Prague, the National Reference Laboratory for Diagnostics of Syphilis, and the National Institute for Public Health, Prague).

All clinical samples were collected after patients gave informed consent. Syphilis was diagnosed based on clinical symptoms and results of several serological tests (e.g. Rapid Plasma Reagin (RPR) test, Venereal Disease Research Laboratory (VDRL) test, *T*. *pallidum* Hemagglutination Assay (TPHA), *T*. *pallidum* Particle Agglutination Assay (TPPA), ELISA IgM and IgG tests and Western blot analyses of IgM and IgG levels). The study was approved by the ethics committee of the Faculty of Medicine, Masaryk University, Czech Republic.

Two types of clinical samples were used for PCR testing, swabs and whole blood samples. Skin and mucosal swabs were transported to the laboratory in a dry state in a sterile capped tube with no fluid transport medium. Whole blood samples (3 ml) were drawn into commercially available containers supplemented with 5.4 mg of K_2_EDTA. Samples collected from Prague’s departments were stored at −20°C and transported on dry ice to the laboratory for PCR testing on bimonthly basis. DNA was extracted within 24 hours after transportation of these samples. Samples from hospitals in Brno underwent DNA extraction within 1–5 days after collection.

Several patients provided two parallel samples, which were obtained during the same physician visit. A combination of two swabs, taken from different sites of the same lesion or from two separate lesions, or a swab and a whole blood sample were obtained from syphilis seropositive patients.

### Isolation and PCR detection of treponemal DNA

Treponemal DNA was isolated as described previously [17] from swabs, which were submerged in 1.5 ml of sterile water and agitated for 5 min at room temperature (0.2 – 0.4 ml of the liquid phase was used for isolation), and from whole blood (0.2 – 0.8 ml) using a QIAamp DNA Mini kit (Qiagen, Hilden, Germany) and the Blood and Body Fluid Spin Protocol. DNA was eluted to 60 μl with AE buffer.

For detection of treponemal DNA in clinical samples, a nested PCR amplification of *polA* (TP0105) and *tmpC* (TP0319) genes was performed as described previously [5,13,17,50].

### Molecular typing of treponemal DNA and DNA sequencing

Treponemal loci (TP0136, TP0548 and 23S rRNA genes) were amplified using nested PCR protocols according to Flasarová et al. [17]. Briefly, each PCR reaction contained 0.5 μl of 10 mM dNTP mix, 2.5 μl of 10× ThermoPol Reaction buffer, 0.25 μl of each primer (100 pmol/μl), 0.05 μl of Taq polymerase (5000 U/ml, New England BioLabs, Frankfurt am Main, Germany), 1 or 10 μl of sample and variable amounts of PCR grade water in 25 μl reactions. PCR amplification was performed at the following cycling conditions: 94°C (1 min); 94°C (30 s), 48°C (30 s), 72°C (60 s), 30 cycles; 72°C (7 min) for TP0136, TP0548 and 23S rRNA genes. The second step of nested PCR was performed under the same conditions, but with an increased number of cycles (40 cycles).

PCR products were visualized with 1.5% agarose gels, purified using a QIAquick PCR Purification Kit (Qiagen, Hilden, Germany) and sequencing was completed using a *Taq* DyeDeoxy Terminator Cycle Sequencing Kit (Applied Biosystems, Foster City, CA, USA). Sequence alignments and assemblies were carried out using the LASERGENE program package (DNASTAR, Madison, USA). For determination of mutations at position 2058 [51,52] and 2059 [53] in the 23S rRNA gene, a *Mbo*II/*Bsa*I restriction digest assay was used [53]. The number of 60-bp repetitions in the *arp* gene was determined as described previously [14,15] and amplification of the *tprE*, *G*, and *J* genes, using nested PCR, was done according to Pillay et al. [15] with two modifications described in Flasarová et al. [17]. The RFLP analysis was carried out according to Pillay et al. [15]. DNA isolated from *T*. *pallidum* strain Nichols (Houston) was used as a control for CDC (*arp*/*tprEGJ*) and sequence-based genotyping (TP0136/TP0548/23S rRNA).

### Statistical methods

Standard methods derived from the binomial distribution, including two-tailed tests were used. An interactive calculation tool for chi-square tests of “goodness of fit” and independence was used [54].

### Availability of supporting data

All sequences identified by sequence-based typing of TP0136, TP0548 and 23S rDNA loci are described in an Additional file 1.

## Competing interests

The authors declare that they have no competing interests.

## Authors’ contributions

LM and PP participated in the study design, carried out PCR testing, analyzed results and prepared a draft version of the manuscript. VW, IK and HZ participated in sample collection and serology testing. DS planned and coordinated the study, and wrote the final version of the manuscript. All authors read and approved the final manuscript.

## Supplementary Material

Additional file 1 Sequence types identified by sequence-based typing. Click here for file
